# Micro- and Nanoscale Imaging of Fluids in Water Using Refractive-Index-Matched Materials

**DOI:** 10.3390/nano12183203

**Published:** 2022-09-15

**Authors:** Shin-ichi Satake

**Affiliations:** Department of Applied Electronics, Tokyo University of Science, 6-3-1 Niijuku, Katsushika-ku, Tokyo 125-8585, Japan; satake@te.noda.tus.ac.jp

**Keywords:** micro digital holographic particle tracking velocimetry, total internal reflection fluorescence microscopy, refractive-index-matching method, nano imprint technology

## Abstract

Three-dimensional (3D) visualization in water is a technique that, in addition to macroscale visualization, enables micro- and nanoscale visualization via a microfabrication technique, which is particularly important in the study of biological systems. This review paper introduces micro- and nanoscale 3D fluid visualization methods. First, we introduce a specific holographic fluid measurement method that can visualize three-dimensional fluid phenomena; we introduce the basic principles and survey both the initial and latest related research. We also present a method of combining this technique with refractive-index-matched materials. Second, we outline the TIRF method, which is a method for nanoscale fluid measurements, and introduce measurement examples in combination with imprinted materials. In particular, refractive-index-matched materials are unaffected by diffraction at the nanoscale, but the key is to create nanoscale shapes. The two visualization methods reviewed here can also be used for other fluid measurements; however, because these methods can used in combination with refractive-index-matched materials in water, they are expected to be applied to experimental measurements of biological systems.

## 1. Introduction

Methods for visualization measurements in fluids include particle image velocimetry (PIV) [1,2] and particle tracking velocimetry (PTV), which measure the flow velocity by capturing the movement of both particles and tracer particles using a high-speed camera. In addition, a method that would enable visualization of the flow around a structure in a fluid as well as the flow behind the structure is strongly demanded. Such a method would facilitate the refractive-index matching (RIM) method [3,4], which can make a structure appear transparent using a combination of a fluid and structure with the same refractive index, thereby preventing refraction and scattering from the structure in the fluid during observations of the structure’s surroundings. Several factors govern the selection of the combination of the object and the hydraulic fluid used in the RIM method. Although numerous methods have been used in combination with the RIM method, using the RIM method in combination with PIV requires a working fluid that meets several requirements. Specifically, the working fluid must (1) not corrode objects, tracer particles, or other components of the experimental system, (2) be easy to handle, exhibiting low toxicity and flammability, (3) exhibit high transmittance at fluorescence wavelengths, and (4) be inexpensive. Depending on the experimental system, it is necessary to prepare several tens to several hundred liters of hydraulic fluid, and the safety and cost aspects (including waste liquid treatment) must be considered. The combination of a sodium iodide (NaI) and acrylic is commonly used because it easily forms structures in fluids during fluid measurements [5]. PIV visualization in a sphere-packed pipe has also been conducted using a matched refractive-index method with NaI solution as the working fluid [6]. In one study, a 30% aqueous solution of potassium hydroxide and a circular pipe made of acrylic were used for PIV measurement [7]. When solution-based working fluids are used, chemical handling becomes difficult and corrosion-resistant equipment is necessary. Therefore, the RIM method using pure water (refractive index 1.33) has become important, and it is necessary to select a material for this purpose that can be used in combination with water and that exhibits a structure compatible with the RIM method.

The purpose of this review is to introduce materials that can be used for measurements in water and to introduce the methods of holographic velocimetry and total internal reflection fluorescence microscopy (TIRFM) to the fluid measurement methods associated with such materials. The polymer-based RIM method for common fluid measurements involving water has recently been reviewed [8]. The resins introduced therein are FEP, THV, MEXFLON, CYTOP, NOA, MY-133-V2000 (MY Polymers, Ltd.). Other materials with a refractive index similar to that of pure water include polyacrylamide hydrogels and agarose hydrogels [9]. Whereas acrylamide is highly toxic, agarose is the main component of agar and is highly safe. However, agarose gel has been reported to be brittle and poorly transparent. MEXFLON has been reported to exhibit enhanced mechanical strength rather than gel-like behavior, and its refractive index is equal to that of pure water [10]. MY-133-V2000 (MY Polymers, Ltd.), a UV-curable resin that is easy to handle and mold, has recently become commercially available. In a study where an index-matching method was conducted using a micropillar and a cell culture solution (*n* = 1.338) produced with one of these resins, MY-134 (MY Polymers, refractive index *n* = 1.34), cells were observed [11]. Section 1 provides an example of a method that uses index materials. Section 2 presents examples of the principles and methods of holographic velocimeters and their applications. Section 3 explains the principles of TIRFM. Section 4 provides an example where TIRFM and the index material MY-133-V2000 were used. In particular, a method to make a film of MY-133-V2000 sufficiently thin for the evanescent wave to reach it is discussed.

## 2. Three-Dimensional Visualization Technique Using Holography in a Microflow

The role of holography-based fluid measurements in the study of fluids has already been reviewed in detail [12]. Holography-based fluid measurements applied within microflows have also been reviewed [13]. In the early stages of the development of holography fluid measurement methods, the particle position was reconstructed from an image acquired by a film plate to identify the particle position and determine the flow velocity [14]. Later, when it became clear that particle images could be captured with digital cameras [15,16,17,18,19,20,21], this technology was quickly applied in fluid measurement in the 2000s [22,23,24,25]. In holography-based fluid measurements, the configuration is classified and described in detail as in-line, off-axis, etc., according to the placement of the optical axis [26]. In this article, we only discuss measurements in the in-line configuration. Historically, camera–object distances have been shortened because of the resolution constraints of camera elements and fluid measurements have been developed in microscopic systems in consideration of the in-line optical axis [27,28,29,30,31]. In particular, our group developed a microdigital holographic particle tracking velocimetry (micro-DHPTV) [32] system and developed a high-speed method [33,34] and hardware [35,36,37] to use the system to measure complex microfluid flow [38] and detect microbubbles [39]. In addition, its accuracy was verified [40] and recently various flow paths were applied [41,42].

**Figure 1:**
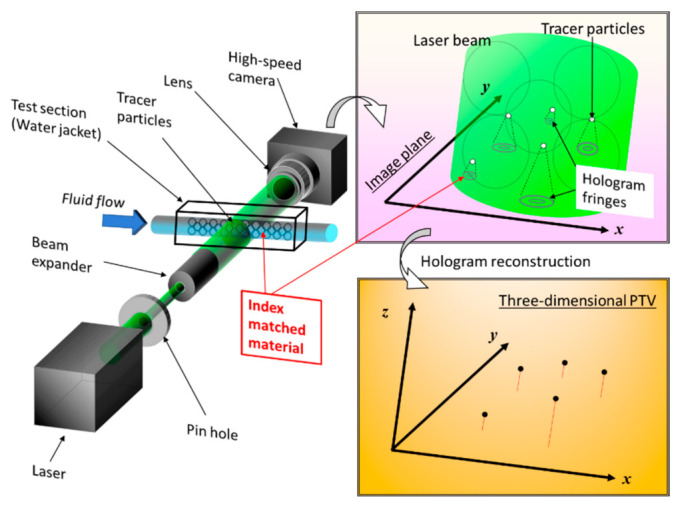
Example of a setting in which DHPTV is applied to an intra-tube flow in a tube filled with MEXFLON pebbles [43].

**Figure 2:**
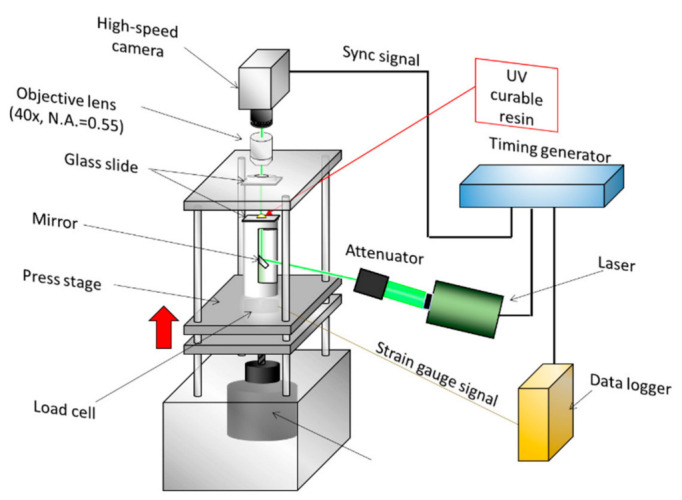
DHPTV setting for squeezed flow in a UV nanoimprint process [44].

The basic principle of hologram recording is explained here using visualization results for the inside of a tube filled with pebbles made of MEXFLON [43] as an example. Figure 1 is a schematic showing the composition of DHPTV characterized by the in-line method. Coherent laser light is used as the light source to form a hologram, which is expanded to the observation area using a beam expander. The magnified laser light passes through the test section containing tracer particles. The X–Y–Z position can be determined simultaneously by reconstructing the obtained hologram image on a computer. When a hologram was reproduced, the fringe image of the particle became the substance at the existing focal length of the particle, but came in a focus, and the image was central, and a hole opened, becoming doughnut-shaped. In addition, it grew large so that the size deviated from the focus, and, seen from the side, it was the form such as the hand drum, the fringe image of the particle in the section. Therefore, at each point of the XY plane, the greatest brightness level from all reproduction side of the point was searched. Then, the point was judged as a particle when the biggest brightness level and brightness level of the neighboring pixels of the point about each point were compared, and the greatest brightness level of the point than the brightness level of the neighboring pixels is big, and the value is bigger than the suitable threshold. The velocity vector of the fluid in the measurement volume can be obtained by detecting the moving quantity of the particle and the direction from the three-dimensional particle coordinate between two times provided by the method mentioned above. Multiple hologram images can be continuously processed, and the flow field can be visualized in three dimensions by PTV. Figure 2 shows measurements of the imprinting process by micro-DHPTV [44]. A technique called UV nano in print lithography (UV-NIL) attracts attention as a core technology of the next-generation semiconductor device process. Ultraviolet rays were irradiated, and this process rigidified resin after transforming a UV-curable resin with a mold. After the resin stiffened, high-speed pattern transcription by separating a mold was performed. However, as in the process of this UV-NIL, lack of filling or lack of hardening of the resin may happen, it was important that the flow of the photocoagulation resin during the process to imprint was measured. Micro-digital holography measurements were performed for the resin crimping process in the imprinting-process apparatus. In the optical axis direction, holographic images were acquired through the crimped glass plates. The flow became a squeezed flow. The flow phenomenon of the UV-curable resin in the imprint process by this distribution was elucidated. By substituting fluorescent particles for the tracking particles in the same macrofluids system, we successfully measured velocity and temperature simultaneously [45]. In recent years, numerous studies have used machine learning to improve the accuracy of holographic processing.

## 3. Three-Dimensional Visualization in a Nanoflow

**Figure 3:**
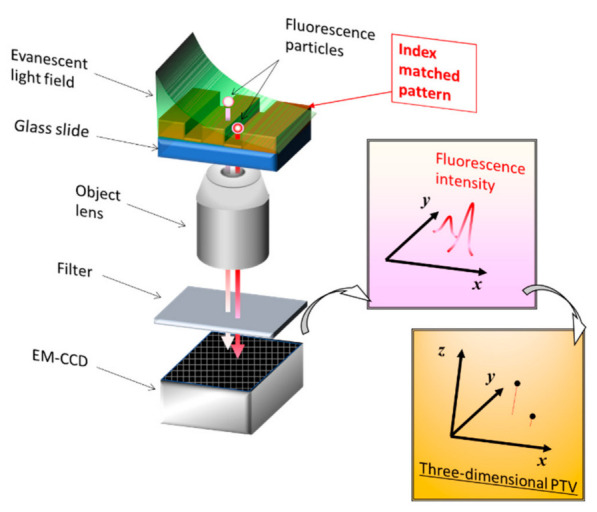
TIRF setting for MnPTV.

In this section, we introduce an example where the RIM method was applied to multilayer nanoparticle image velocimetry (MnPIV). The evanescent-light-seeping principle was applied to a microscope system [46,47], and fluorescent particles were inserted into the system to track its movement via flow velocimetry [48]. Super-resolution measurement methods, including the total internal reflection technique, have already been reviewed [49]. The particle tracking method uses the aforementioned principles of PIV and PTV. We first explain the principle of three-dimensional (3D) visualization of evanescent light (Figure 3). Laser light is injected into the system through a glass slide or similar substrate. When the system is irradiated under total internal reflection (TIR) conditions, evanescent light is generated on the opposite side. This evanescent light is used as a light source to illuminate the fluorescent particles. Because the intensity of the generated evanescent light decreases exponentially when it leaves the substrate, only the polar vicinity of the substrate is illuminated brightly.

In the MnPTV method, the height position is obtained from the emission intensity of the fluorescent particles illuminated in this region of the substrate. For observation, calibration of the attenuation curve in advance was necessary, and the IM method was used for this calibration. A deformed MgF_2_ nanoscale thin film and 1-propanol were used [48].

In addition, incident radiations are scattered near the edge of the pattern by two difference in refractive index of materials when it is a combination of water and polydimethylsiloxane (PDMS) [50].

## 4. Imprint Technology for Nano-Obstacles for TIRF Measurements

This section introduces a method for creating calibration plates for TIRFM. Because both methods enable the RIM method under TIRFM, the purpose is to form a material pattern having a refractive index of 1.33 in the form of a glass slide. The most important consideration here is to create an accurate nanoscale stepped structure because the penetration depth of evanescence light is on the order of several hundred nanometers.

**Figure 4:**
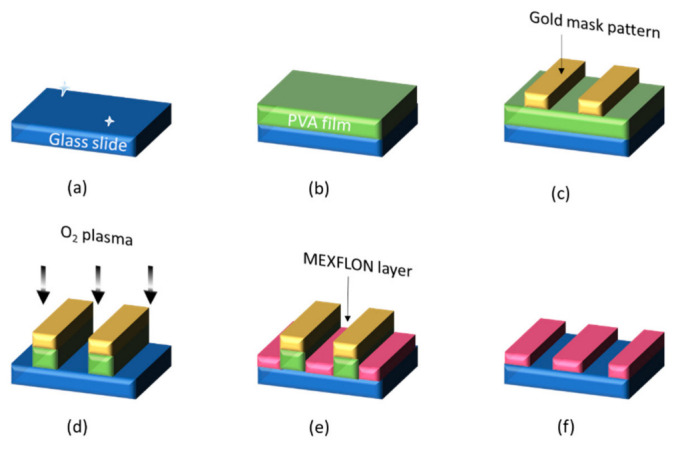
Fabrication process for nanosteps using IM material via vacuum deposition and lift-off: (**a**) cleaning; (**b**) spin-coating; (**c**) metal mask by nTP; (**d**) etching; (**e**) deposition; (**f**) lift-off by water.

The first method is the same method used in conventional multilayer-PIV [51], where thin films are prepared using vacuum deposition. Figure 4 shows how to create it. The glass slide is first cleaned by ultrasonication (a), and then a polyvinyl alcohol (PVA) layer is deposited by spin coating (b). Nanoprinting techniques are then used to transfer gold mask patterns onto the PVA [52]. The PVA layer is etched with oxygen plasma to partially expose the glass slide surface (d). MEXFLON, which is an IM material, is then vacuum-deposited (e). Finally, the PVA layer is dissolved with water and the target MEXFLON layer is obtained via a lift-off step. The advantage of this method is that it enables the film thickness to be controlled with high precision (~10 nm). However, three vacuum processes are required during the vapor deposition of gold and MEXFLON and also during oxygen plasma etching, which is relatively complicated (time consuming). Only one height can be calibrated with a single calibration plate [53].

**Figure 5:**
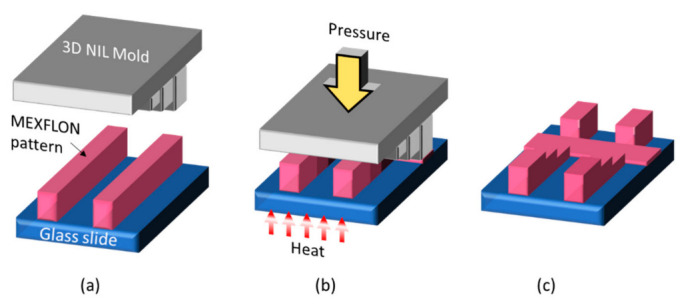
Three-dimensional calibration-plate fabrication process for TIRFM with RIM: (**a**) a nanoimprint mold having a 3D structure is positioned; (**b**) thermally nanoimprinted; (**c**) a MEXFLON pattern having a 3D structure after cooling to room temperature.

A method has also been developed in which a 3D thermal nanoimprint is used in conjunction with a single calibration plate to enable multipoint height calibration [54]. In Figure 5, a nanoimprint mold having a 3D structure is positioned (a) and thermally nanoimprinted (b) with respect to the MEXFLON pattern created by the previous method. Thereafter, to create a calibration plate more easily by peeling off the mold after it has cooled to room temperature, a second method using thermal nanoimprinting is applied. Here, MEXFLON, an IM material, is initially vacuum-deposited onto the glass slide (a) and then the microstructure is patterned by thermal nanoimprinting (b). Finally, when the mold is peeled off after cooling to room temperature, a MEXFLON pattern having a 3D structure is obtained (c). Here, only the highest part of the mold pushes the MEXFLON layer apart; however, other lower, uneven parts can be deformed to follow the original pattern. The surface on which the glass slide is exposed remains intact and can be set as the zero point at the time of brightness calibration, and the height point of multiple stages can be calibrated simultaneously. As a result, the calibration-plate replacement time at the time of TIRF calibration is substantially shortened.

**Figure 6:**
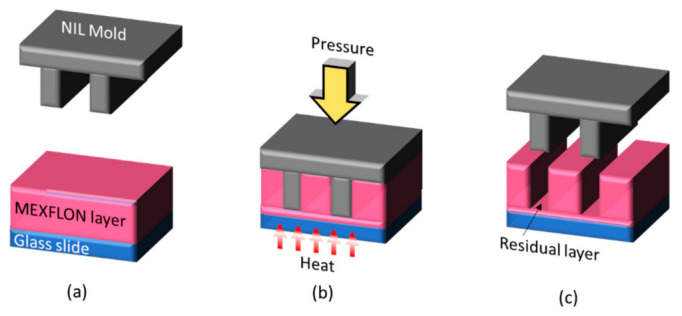
Micropattern fabrication process with a RIM material using thermal nanoimprint lithography for TIRFM: (**a**) deposition; (**b**) thermal nanoimprint; (**c**) cooling and releasing.

Figure 6 shows a method for creating an observation plate for observing the movement of fluid near a microstructure by TIRFM using the RIM method [55]. In this method, MEXFLON, which is a RIM material, is vacuum-deposited onto the entire surface of the glass slide in advance (a). The MEXFLON film thickness at this time needs to be thinner than the seeping depth of the evanescent light. Thereafter, a nanoimprint mold having a microstructure is used to thermally nanoimprint the MEXFLON layer (b). Finally, after the plate reaches room temperature, the mold is peeled off and the microstructure is transferred to the MEXFLON layer (c). The MEXFLON pattern created in this manner is RIM in water; thus, TIRFM observations can be conducted as usual. This method has opened a path to visualizing the flow around a microstructure in detail.

**Figure 7:**
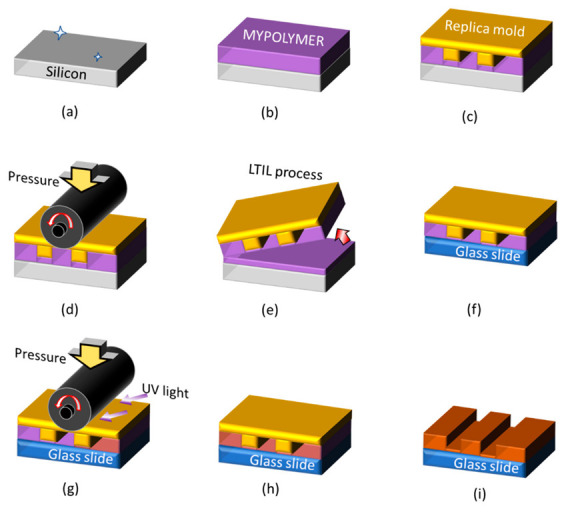
Micropattern fabrication process with RIM material using UV nanoimprint lithography and LTIL for TIRFM: (**a**) cleaning; (**b**) spin-coating; (**c**) positioned; (**d**) first press by a roll, (**e**) first release from one side (i.e., LTIL); (**f**) positioned; (**g**,**h**) UV curing is performed while the film thickness is increased to the final target value again by a roller-imprinting device film; (**i**) release.

**Figure 8:**
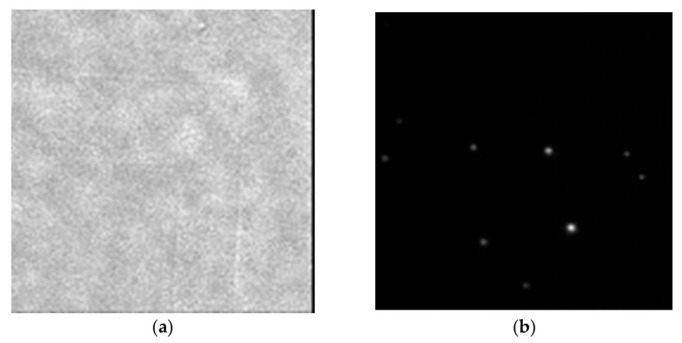
Florescent particles on a MY-133-V2000 pattern: (**a**) lamp lighting without water; (**b**) under evanescent lighting with water.

Characterizing residual films using conventional methods is difficult because the influence of the bending transformation of the mold when a structure is transferred onto the glass used in TIRM must be considered [56,57]. Therefore, techniques that generally realize a free residual layer using a flexible mold have been developed [58,59]. However, roll-type liquid-transfer imprint lithography (LTIL) is useful for MY-133-V2000, which is a refractive-index-matched material with high viscosity, which enables the fabrication of a thinner residual film. Therefore, we developed a fabrication method for creating RIM plates for TIRFM via a method that does not involve a vacuum process [60,61]. The most important feature of this method is the use of MY-133-V2000, which is a photocurable resin with a refractive index equal to that of water. If there is a mold with a microstructure, it can be created at room temperature and under atmospheric pressure. However, because MY-133-V2000 is a highly viscous liquid, ordinary optical nanoimprinting (UV-NIL) cannot easily make the film under the pattern (known as the residual film) thinner than the evanescent light seeping depth. Therefore, Figure 7 shows a process where LTIL technology is used to overcome this problem. First, a silicon wafer with a mirror surface is cleaned (a) and MY-133-V2000 is formed on it by spin coating (b). The pre-created replica mold is positioned (c), the thickness of the liquid film of MY-133-V2000 is reduced using a roller imprinting device (d), and then the replica mold is peeled off from one side and an LTIL process is performed to form a nano-thin film of MY-133-V2000 on the mold (e). A replica mold in which MY-133-V2000 is formed is positioned on the glass slide (f), and UV curing is performed while the film thickness is increased to the final target value again by a roller-imprinting device (g–h). Finally, the replica mold is peeled off (i). With this LTIL method, even a liquid material with a high viscosity can limit the thickness of the remaining film to approximately several tens of nanometers or less. The resultant MY-133-V2000 membrane can also be used for TIRFM calibration and visualization of flow around microstructures. Figure 8 shows MY-133-V2000 with a calibration step, prepared by this method. The left figure is a lamp image with a shadow of a step; the right figure is a fluorescence image. No step effect is observed on the fluorescent particle image. This approach enabled visualization of the movement across the step by capturing the Brownian motion with a plate produced by the same method [62,63].

## 5. Conclusions

In the first half of this paper, we introduced DHPTV as a method for visualizing the flow field using the index-matching method. Measurements from macroscale to microscale order are possible. In the second half of this paper, MnPTV was introduced as a technology that applies the RIM method as a nanoscale approach. The combination of a thin film prepared using MY-133-V2000 and water enables visualization in water without using an MgF_2_ film and a general pair such as 1-propanol in MnPTV. We believe that these technologies that can visualize liquids that contain water will be a powerful tool for clarifying details of liquid flow in the biotechnology field.

## Data Availability

Not applicable.
